# Exosomes: vesicular carriers for intercellular communication in neurodegenerative disorders

**DOI:** 10.1007/s00441-012-1428-2

**Published:** 2012-05-19

**Authors:** Anja Schneider, Mikael Simons

**Affiliations:** 1 Department of Psychiatry and Psychotherapy, University Medicine Goettingen, Von-Siebold-Str.5, 37075 Goettingen, Germany; 2 DFG Research Center for Molecular Physiology of the Brain, CMPB, Goettingen, Germany; 3 German Center for Neurodegenerative Diseases (DZNE), Goettingen, Von-Siebold-Str.5, 37075 Goettingen, Germany; 4 Max-Planck-Institute for Experimental Medicine, Hermann-Rein-Str.3, 37075 Goettingen, Germany; 5 Department of Neurology, University Medicine Goettingen, Robert-Koch-Str. 40, 37075 Goettingen, Germany

**Keywords:** Exosomes, Dementia, Spreading, Transfer, Aggregopathy

## Abstract

The intercellular transfer of misfolded proteins has received increasing attention in various neurodegenerative diseases characterized by the aggregation of specific proteins, as observed in Alzheimer’s, Parkinson’s and Huntington’s disease. One hypothesis holds that intercellular dissemination of these aggregates within the central nervous system results in the seeded assembly of the cognate soluble protein in target cells, similar to that proposed for transmissible prion diseases. The molecular mechanisms underlying the intercellular transfer of these proteinaceous aggregates are poorly understood. Various transfer modes of misfolded proteins including continuous cell-cell contacts such as nanotubes, unconventional secretion or microvesicle/exosome-associated dissemination have been suggested. Cells can release proteins, lipids and nucleic acids by vesicular exocytosis pathways destined for horizontal transfer. Encapsulation into microvesicular/exosomal vehicles not only protects these molecules from degradation and dilution in the extracellular space but also facilitates delivery over large distances, e.g. within the blood flow or interstitial fluid. Specific surface ligands might allow the highly efficient and targeted uptake of these vesicles by recipient cells. In this review, we focus on the cell biology and function of neuronal microvesicles/exosomes and discuss the evidence for pathogenic intercellular protein transfer mediated by vesicular carriers.

## Cell biology of microvesicles and exosomes

Microparticles have been isolated from various body fluids such as urine, ascites, saliva, breast milk and blood by ultracentrifugation, ultrafiltration or immunoprecipitation (Simpson et al. 2009). A consensus regarding the nomenclature of these heterogeneous vesicular populations is still missing because of experimental difficulties in separating and distinguishing the various extracellular vesicles based on their biochemical or morphological properties. The terminology mainly refers to the cellular origin (e.g. aggrosomes, prostasomes, prominosomes), their attributed function (e.g. apoptotic body), size (ranging from 40 nm to 4 μm) or subcellular origin (exosomes, shedding vesicles; see Table 1). Whereas exosomes are built within the endosomal system, shedding vesicles (or ectosomes) bud directly from the plasma membrane into the extracellular space. Shedding vesicles can be further divided into microvesicles, with variable diameters of 0.1 to 1 μm and the larger apoptotic bodies.

**Table 1:** Extracellular vesicles and their characteristics

Microparticles		Origin	Size	Flotation
Exosomes		Multivesicular endosome	40–100 nm	1.13–1.19 g/ml
Shedding vesicles (ectosomes)	Microvesicle	Plasma membrane	0.1–1 μm	
	Apoptotic body	Plasma membrane	1–4 μm	1.24–1.28 g/ml

### Exosomes

Exosomes are generated within the (late) endosomal compartments by inward vagination and fission of the limiting membrane. Endosomes that are filled with these intraluminal vesicles (ILV) are termed multivesicular endosomes (MVE). ILVs can serve as storage compartments for proteins and signalling complexes and can re-enter the cytosol by backfusion with the MVE limiting membrane (Abrami et al. 2004; Le Blanc et al. 2005; Dobrowolski and De Robertis 2011). In addition to a mere storage function, the MVE can either fuse with the lysosome, followed by the degradation of ILVs, or with the plasma membrane to release the ILVs as exosomes into the extracellular space. Whether these different pathways correspond to distinct subclasses of MVEs or whether each MVE can switch between the different itineries described above is unknown. Exosomes contain cytosol and feature a membrane topology that is inverse to the endosomal membrane. The inner exosomal membrane leaflet faces the cytosol, whereas the outer leaflet adjoins the extracellular space. Exosomes are secreted by a variety of cells in vitro and in vivo under physiological and pathological conditions. On transmission of electron or cryo-electron microscopic images, exosomes appear as vesicles of 40–100 nm in diameter with a characteristic round or cup-shaped morphology (Thery et al. 2006; Conde-Vancells et al. 2010). Exosomes differ in their origin and in their protein and lipid composition. Depending on their cellular ancestry, they carry cell-type-specific proteins, such as major histocompatibility complex (MHC) when released from antigen-presenting cells, or myelin proteins, when derived from oligodendrocytes (Kramer-Albers et al. 2007; Thery et al. 2001). Several proteins are specifically enriched in exosomes and serve as marker proteins. These include the integrins and tetraspanins CD63, CD89, CD81, CD9 and CD82, the MVE proteins alix and tsg101, the endosomal and endosome maturation-related proteins flotillin and annexin and the heat shock proteins hsp70 and hsp90 (Simons and Raposo 2009). Proteins derived from the nucleus, mitochondria or endoplasmic reticulum are mainly excluded from the exosomal pathway.

### Shedding vesicles

Shedding vesicles (or ectosomes) are generated by shedding at the plasma membrane and include microvesicles with a heterogeneous size range from 100 nm to 1 μm and apoptotic bodies. Apoptotic bodies are released from the plasma membrane during the breakdown of apoptotic cells. They carry DNA, histones, organelles and surface markers that allow their recognition and internalization by phagocytic and other subsequent cells, thereby preventing the release of intracellular content and inflammatory reactions (Nunez et al. 2010; Thery et al. 2001). Their diameter varies between 1 and 4 μm. Shedding particles with a diameter of 100 nm cannot be distinguished from endosomally derived exosomes on a morphological or biochemical basis, including density gradient centrifugation. Some authors refer to these vesicles as exosomes derived from the direct pathway as compared with exosomes that stem from the endosomal indirect pathway (Booth et al. 2006; Simons and Raposo 2009). Further complexity is added by the finding that several proteins can bud either into exosomes or shedding vesicles in a cell-type-dependent manner (Shen et al. 2011a). Throughout our review, we will therefore use the umbrella term “exosomes and microvesicles” (EMV) to describe extracellular vesicles that are of 40-100 nm in size and that are generated within both pathways as suggested by Shen et al. (2011a). Despite the experimental difficulties in distinguishing between exosomes and microvesicles, they might still represent distinct entities with different properties and functions.

### Physiological function of EMVs

Exosomes were first identified as a pathway for shuttling superfluous material out of the cell, especially from cells with low lysosomal activity or lysosome number. Only recently has their role as an alternative exocytosis pathway for cytosolic or transmembrane proteins and their function in the targeted delivery of molecules destined for intercellular communication and signalling been recognized (Mathivanan et al. 2010b). Targeting mechanisms for the selective sorting of proteins, lipids, mRNA and small non-coding RNA are under intense investigation since certain cellular subsets of these molecules are specifically enriched in EMVs. There is ample evidence for a role of EMVs in intercellular communication; however, the mechanisms for target cell recognition, entry and the intracellular itinery in recipient cells are far from being understood. Regarding the central nervous system (CNS), EMV release has been shown in vitro for oligodendrocyte, microglia, astrocyte and neuronal cell cultures (Faure et al. 2006; Kramer-Albers et al. 2007; Potolicchio et al. 2005; Taylor et al. 2007).

## Neuronal EMVs

### Origin

Primary neurons release vesicles which can be isolated from conditioned medium in vitro (Faure et al. 2006). Their size and morphology as assessed by gradient centrifugation and electron microscopy closely resemble EMVs and the preparations are positive for exosomal marker proteins, such as hsp70 and flotillin (Bulloj et al. 2010; Faure et al. 2006; Lachenal et al. 2011). Because of the lack of specific exosomal marker proteins, difficulties abound when trying to establish whether these vesicles represent bona fide exosomes derived from the indirect endosomal pathway. Recently, Lachenal et al. (2011) have demonstrated the presence of tetanus toxin in EMV preparations derived from neuronal culture medium. Tetanus toxin is endocytosed from the cell surface and is present in endosomes. The authors therefore speculate that these tetanus-toxin-positive EMVs originate from the indirect pathway (Lachenal et al. 2011). However, the presence of tetanus toxin does not exclude direct budding from the plasma membrane, since tetanus toxin primarily binds to membrane gangliosides and would also be expected in vesicles that bud directly from the plasma membrane.

Neuronal MVEs are predominantly distributed within the somatodendritic compartment where they are 50 times more abundant than in the axon (for a review, see Von Bartheld and Altick 2011). The accumulation of MVEs at the postsynapse indicates that MVE fusion and exosome release might occur from dendritic spines. Electron-microscopic examination of stimulated primary neuronal cultures has demonstrated vesicular structures with the size and morphology of exosomes in close proximity to somatodendritic compartments (Lachenal et al. 2011). More experiments, e.g. with chamber systems, are needed to improve the characterization of the sites of EMV release in polarized neurons. In addition, knowledge of whether MVEs released from different neuronal subcompartments are distinct with regard to their molecular composition and cargo would be of interest.

### Function

Neuronal MVEs have been shown to carry glutamate receptor (GluR2) subunits. MVE-mediated release could therefore be a mechanism to eliminate α-amino-3-hydroxy-5-methyl-4-isoxazoleproprionic acid (AMPA) receptors in response to glutamatergic stimulation (Lachenal et al. 2011). Thus, exosomes released from the postsynaptic site might modulate synaptic transmission and plasticity. This notion is further supported by the finding that the number of dendritic MVEs and EMV release increase in electrically stimulated neurons (Kadota et al. 1994; Kraev et al. 2009). Likewise, prolonged potassium-induced depolarization of neuronal cultures potentiates EMV secretion (Faure et al. 2006). Further evidence for activity-dependent EMV release has been provided by Lachenal et al. (2011) who have demonstrated that neuronal EMV secretion is regulated by calcium influx and glutamatergic activity. Not only treatment with ionomycin to raise intracellular calcium concentrations but also increased glutamatergic activity after pharmacological inhibition of γ-aminobutyric acid (GABA)-A receptors results in enhanced EMV secretion from neuronal cultures. Interestingly, treatment with AMPA- or N-methyl D-aspartate (NMDA)-receptor antagonists counteract the glutamatergic effect on EMV release. Hence, the authors speculate that neurons modulate their number of ionotropic postsynaptic receptors, synaptic plasticity and strength by activity-dependent EMV release (Lachenal et al. 2011).

In vivo evidence of neuronal exosome release and its functional significance is still lacking. The transduction of wnt signalling by exosome-like vesicular structures has been reported in *Drosophila*. The palmitoylated wnt proteins are membrane-bound and thus unlikely to be released as soluble proteins to the extracellular space. Instead, the *Drosophila* wnt1 homolog wingless (wg) has been shown to be transported trans-synaptically with vesicles resembling exosomes, followed by the binding of wg to *Drosophila* frizzled 2 (DFz2) receptors at the postsynapse (Korkut et al. 2009). Further in vivo evidence for neuronally derived EMVs is based on their presence in cerebrospinal fluid (CSF). Vella et al. (2008) have described the isolation of microparticles, which are enriched in the native prion protein PrPc, from ovine CSF. Harrington et al. (2009) have identified, in human CSF, nanostructures including exosome-like vesicles that can be labelled with antibodies against various exosomal marker proteins in immuno-transmission electron microscopy. Whereas these vesicles might be derived from CSF immune cells or ventricular ependymal cells, we have been able to fractionate, from human CSF, exosome-shaped vesicles positive for GluR2, indicating their neuronal origin (own unpublished data).

## Exosomes in neurodegenerative diseases

Although definitive evidence for intercellular EMV transfer within the CNS is still lacking, EMVs have been repeatedly discussed as potential carriers in the dissemination of disease pathology in neurodegenerative disorders (for a review, see Aguzzi and Rajendran 2009).

### Prions

This hypothesis evolved first in the context of the interneuronal spreading of transmissible prion disorders such as the new variant of Creutzfeld-Jacob disease (CJD), bovine spongiform encephalitis (BSE) and scrapie. Prions exist in two different conformational states: the natively folded PrP^c^ and the disease-associated misfolded PrP^sc^. PrP^sc^ is characterized by an abnormal conformation, which can serve as a template to induce the misfolding of PrP^c^ (a mechanism called permissive templating). In infectious prion diseases, PrP^sc^ can enter the organism by the gut, followed by the invasion of lymphoid tissue from where it spreads into the peripheral nervous system and finally the CNS. In addition to intercellular transfer by tunneling nanotubes, as discussed by Gousset et al. (2009), a role for exosomes as a carrier for PrP^sc^ in this intercellular dissemination has been proposed. Tunnelling nanotubes are transient membranous connections that can connect cells over distances of up to 100 μm. Two types of nanotubes can be distinguished based upon their diameter and cytoskeleton, which includes either actin or actin and microtubules. The transport of vesicles and organelles has been demonstrated within nanotubes that can bridge the distance between numerous cell types (Gurke et al. 2008). PrP^sc^-bearing exosomes can travel either with the blood stream or after internalization within blood cells to reach their target cells. This hypothesis has been triggered by the finding that cell culture medium from a scrapie-infected hypothalamic GT1 cell line can induce PrP^sc^ formation in recipient cells, indicating a cell-free transfer mode (Schatzl et al. 1997). Both PrP^c^ and PrP^sc^ are released from cells expressing ovine PrP together with vesicles that, based on their morphology, biochemical properties and protein composition, closely resemble exosomes (Fevrier et al. 2004). Exosomal PrP^sc^ and PrP^c^ secretion from an endogenously PrP-expressing neuronal cell line has been reported upon infection with PrP^sc^ (Veith et al. 2009; Vella et al. 2007). Incubation of target cells with exosome preparations from prion-infected neuronal cells is sufficient to induce the conformational shift to PrP^sc^ in various target cell lines. Furthermore, intracerebral injection of PrP^sc^-positive exosomal membranes triggers neurodegeneration and death in recipient mice transgenic for ovine PrP (Fevrier et al. 2004). Both PrP^c^ and PrP^sc^ have been detected in late endosomes and MVEs on an ultrastructural level, indicating an exosomal pathway (Ersdal et al. 2009; Godsave et al. 2008; Laine et al. 2001; Marijanovic et al. 2009).

The subcellular compartment in which the conformational shift from PrP^c^ to PrP^sc^ takes place remains unclear; however, speculation that the MVE/EMV system is involved via local protein enrichment, favourable pH and the lipid environment is tempting. Macromolecular crowding has been shown to promote the conversion to β-sheet structure and the oligomerization of prions (Huang et al. 2010). Exosomal enrichment of PrP^c^ and PrP^sc^ might generate a high local concentration and close proximity between template and PrP^c^, thereby facilitating the conformational shift to PrP^sc^. Furthermore, the conversion of PrP^c^ to PrP^sc^ requires the partitioning of PrP into sphingolipid- and cholesterol-rich membrane domains, which are present in exosomal membranes (Baron et al. 2002; Laulagnier et al. 2004; Subra et al. 2007). Along this line, the in vitro generation of infectious PrP^sc^ from bacterially expressed recombinant PrP^c^ has been shown to require the presence of lipid cofactors, such as the synthetic anionic phospholipid POPG (1-palmitoyl-2-oleoylphosphatidylglycerol; Wang et al. 2010). In addition, several studies have indicated that conversion takes place in acidic endosomal compartments, arguing again for a conversion within the late endosome/MVE (Peters et al. 2003). Alternatively, the fusion of PrP^sc^-positive exosomes with the recipient cell membrane might induce the conversion of PrP^c^ at the target cell surface, as has been indicated by Baron et al. (2002) who have shown that the conversion of PrP^c^ to PrP^sc^ requires the insertion of PrP^sc^ into target cell membranes and the formation of a contiguous membrane layer.

### AA-amyloidosis

Similar to transmissible prion diseases, an exosome-mediated transfer of misfolded proteins has been shown for systemic AA-amyloidosis in vivo. Serum amyloid-A (SAA) proteins are apolipoproteins that are expressed in the liver and that circulate in the blood stream bound to high density lipoproteins. Under inflammatory conditions and interleukin-1 and -6 and tumor necrosis factor stimulation, the expression of these acute phase proteins is increased up to 1000-fold. During chronic inflammation such as rheumatoid arthritis, high concentrations of SAA eventually lead to the formation of a nucleus and polymerization of otherwise soluble SAA proteins into amyloid fibrils. Deposits of SAA fibrils can be found in the interstitial space of many organs. Similar to prion protein misfolding, this SAA fibrillation involves a conformational shift of SAA protein into a β-sheet structure followed by aggregation. Mouse models of experimental AA-amyloidosis develop systemic amyloid deposits under chronic inflammatory conditions triggered by the intravenous, intraperitoneal or oral application of SAA-containing tissue or circulating blood monocytes derived from murine SAA mouse models. This process is reminiscent of transmissible prion diseases (Axelrad et al. 1982; Werdelin and Ranlov 1966). The “seeding” factor, also termed amyloid-enhancing factor (AEF), has been shown to consist in either SAA oligomers or SAA fibrils (Lundmark et al. 2002; Senthilkumar et al. 2008; Sponarova et al. 2008). Tasaki et al. (2010) have demonstrated that blood and plasma derived from experimental murine SAA amyloidosis models can induce pathology in recipient animals and that freeze-thaw cycles abolish the seeding activity of these plasma samples. The authors have been able not only to show that plasma EMVs isolated from mice with SAA amyloidosis carry oligomeric and prefibrillar SAA but also that these EMVs are sufficient to transmit disease pathology to recipient animals (Tasaki et al. 2010). Noteworthy, though, is that not all exosome preparations possess seeding capacity, which might be a result of shearing forces or the clumping of EMVs during the preparation process. Another possible explanation is that only EMVs derived from SAA-positive organs can induce amyloidosis in recipient mice and that these EMVs are not present in the plasma in sufficiently high numbers all the time.

An oral transmission of SAA amyloidosis among cheetahs, which secrete SAA fibrils in their faeces, has been reported (Zhang et al. 2008). Potentially infectious SAA fibrils have also been detected in foie gras (Solomon et al. 2007). Several lines of evidence point to an uptake of exogenous SAA amyloid seeds via the epithelial cells in Peyer’s plaques, followed by transepithelial transport, internalization into follicular dendritic cells and transfer to the spleen where amyloid replication and deposition occurs (for a review, see Westermark and Westermark 2009). This itinery most likely reflects a selective targeting pathway rather than random uptake and release of free circulating fibrils. The exosomal transfer of SAA aggregates could help to explain this reproducible route of seed propagation attributable to tissue- or cell-specific uptake signals on the surface of EMVs. Similar to transmissible prion diseases, cells from the lymphomoncytic lineage could mediate amyloid transport by the uptake of SAA-positive EMVs via specific receptors, followed by transport within the circulation and release through another round of exocytosis in the target tissue. In support of this notion, macrophages have been shown in vitro to be able to internalize AEF from the culture medium and SAA has been detected in various endocytic compartments (Kluve-Beckerman et al. 2001). Immunoelectron analysis has revealed fibrillar SAA protein in lysosomes and LAMP-positive structures in monocytoid cells from SAA amyloidosis mice (Chronopoulos et al. 1994). Taken together, these findings are compatible with endocytic uptake, transport with the blood stream and the exocytosis and transfer of SAA aggregates via the MVE/EMV pathway.

### Neurodegenerative aggregopathies

The transneuronal spreading of oligomers or fibrillar aggregates is increasingly recognized in a variety of neurodegenerative disorders including tau protein and amyloid-β peptide in Alzheimer’s disease, superoxide dismutase 1 (SOD1) in amyotrophic lateral sclerosis (ALS), huntingtin in Huntington’s disease (HD) and α-synuclein in Parkinson’s disease (PD). Aggregopathies do not belong to the class of prion diseases, since infectious transmission between two individuals has never been observed. However, intra- and inter-individual spreading of disease pathology in several of these aggregopathies has led to their classification as possible prionoid disorders (Aguzzi and Rajendran 2009). Strikingly, all proteins involved in the pathogenesis of these diseases seem to be present in EMVs.

### α-Synuclein

**Fig. 1:**
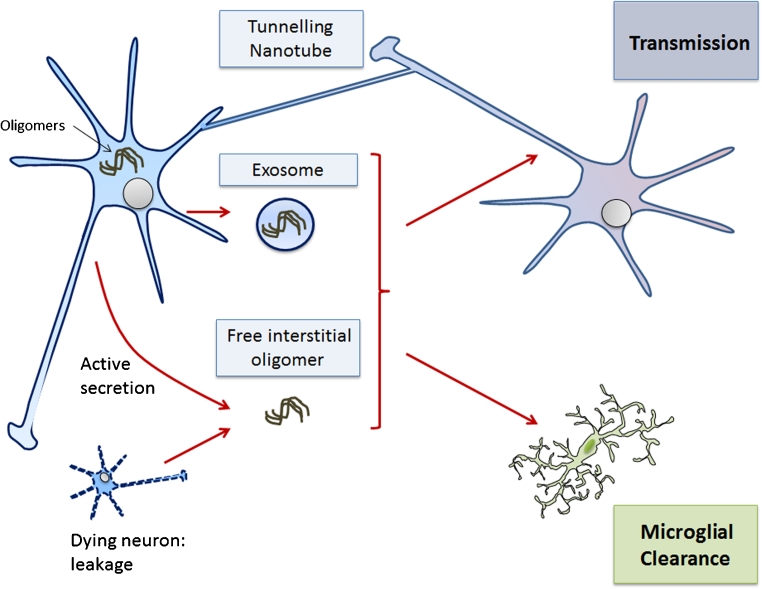
Mechanisms of intercellular transfer of aggregates in neurodegenerative disorders. Misfolded proteins could either be transported via tunnelling nanotubes between cells, within EMVs or by unconventional secretion of free protein. Extracellular misfolded protein moieties could be cleared by the microglia or internalized into neurons where they might serve as seeds to induce protein aggregation

PD is characterized by intracellular aggregates of α-synuclein, which are refered to as Lewy bodies. Lewy bodies appear first in the brainstem followed by the subsequent deposition of aggregates in higher brain regions. The spatial distribution of Lewy body pathology over time follows a predictable anatomical course that reflects patterns of neuronal connectivity (Braak et al. 2004). Similarily, anatomically connected spreading patterns have been observed in prion models of the Syrian hamster after the oral uptake of prions, starting in the dorsal vagus nerve and followed by the medulla, pons, midbrain and cerebellum (Natale et al. 2011). Likewise, after the injection of infectious prions into the eye, the pathology develops along the optical tracts (Liberski et al. 1990). The hypothesis of interneuronal disease propagation in synucleinopathies has been fuelled by the finding that transplanted fetal neurons in PD patients accumulate intraneuronal α-synuclein aggregates, indicating a possible transfer of pathology from substantia nigra host neurons to grafted striatal neurons (Kordower et al. 2008; Li et al. 2008). In a similar fashion, host to graft transmission of α-synuclein has been observed in an α-synuclein transgenic mouse model in which green-fluorescent-protein-labelled neuronal stem cell transplants incorporate the host’s transgenically expressed α-synuclein (Desplats et al. 2009). The induction of α-synuclein aggregation and the worsening of behaviour and/or motor phenotype have been demonstrated in transgenic mice after the intracerebral injection of brain extracts derived from older littermates that exhibited α-synuclein aggregates (Mougenot et al. 2011). Interneuronal transfer of α-synuclein aggregates could serve as a seed to induce aggregation in the host neuron and contribute to the dissemination of aggregates throughout the brain, similar to prion-like self-propagation. Intercellular transfer and the induction of disease pathology have recently been described for PrP^sc^. Intercellular propagation of α-synuclein seeds could either be mediated by tunnelling nanotubes, which connect neighbouring neurons, by extracellular α-synuclein species passively released from dying neurons or by active secretion, including EMV-based release (Fig. 1; Agnati et al. 2010; Danzer et al. 2011; Emmanouilidou et al. 2010). An atypical secretion mechanism has been discussed, as has passive release from dying neurons, to explain the extracellular presence of this cytosolic protein, which lacks conventional secretion signals. Extracellular non-vesicular α-synuclein has been detected in tissue culture medium and in CSF and its concentration is increased under cellular stress conditions suggesting a regulated release mechanism (Jang et al. 2010). In addition, α-synuclein has been demonstrated in EMVs derived from neuronal cultures (Emmanouilidou et al. 2010). To date, the form of extracellular α-synuclein that is relevant for the disease pathology and the way that the cytosolic protein can be actively secreted from cells are unknown. EMVs could act as “Trojan horses” in the transneuronal propagation of α-synuclein aggregates (Brundin and Olsson 2011). Speculation that α-synuclein-containing EMVs are internalized into target cells at a much higher efficiency than non-vesicular α-synuclein species is tempting. In addition, the exosomal compartment could favour the aggregation of α-synuclein by increased local protein concentrations, pH and high membrane curvature, similar to the situation that we discussed for the case of prion protein transconformation. Aggregates of α-synuclein are well established to be able to act as seeds to trigger the aggregation of the monomeric protein. For example, Hansen et al. (2011) have demonstrated cellular release, endocytic uptake, co-dimerization and aggregate formation of α-synuclein in recipient cells within a co-culture system. The transfer of α-synuclein is independent of direct cell-cell contacts; however, despite the presence of α-synuclein in EMVs, they have yet to be shown to be the carriers for intercellular α-synuclein transfer. In vivo evidence of a functionally active uptake of exosomes into postmitotic neurons has recently been provided by Alverez-Eviti et al. (2011), although only with exosomes that have been produced in transgenic cells that transgeneously express a rabies glycoprotein construct that is sorted into exosomes and confers neuroglia-specific uptake. Alternatively, α-synuclein might reach the target cell upon unconventional secretion or passive release from dying cells (Nickel and Rabouille 2009). The trans-synaptic transmission of toxic α-synuclein oligomers has been demonstrated in tissue culture models (Danzer et al. 2011). The proportion of extracellular α-synuclein that is localized in EMVs and the form (free or EMV-encapsulated) of α-synuclein that confers toxicity and/or seeding capacity remain unknown.

### Tau

In AD and other tauopathies such as corticobasal degeneration, progressive supranuclear palsy and a subgroup of frontotemporal dementias, intracellular aggregates of the microtubule-associated protein tau are assumed to mediate neuronal dysfunction and subsequently neurodegeneration. Tau aggregates in AD emerge first in the entorhinal cortex followed by propagation to hippocampal regions, temporal lobes and more distant neocortical areas (Bancher et al. 1993). Recently, the interneuronal transmission of tau pathology was reported in vitro whereby exogenously added tau fibrils were internalized into host cells and induced the aggregation of endogenous tau protein (Frost et al. 2009; Guo and Lee 2011). In addition, tau aggregates have been shown to be transferred between cells in a co-culture system (Clavaguera et al. 2009). Similar results have been obtained in vivo in mice that express a human wild-type tau transgene and that do not develop tau filaments under normal conditions (Clavaguera et al. 2009). Here, the intracerebral injection of brain extracts derived from mutant P301S tau transgenic mice leads to the aggregation of wild-type human tau in host mice. The seeding capacity is dependent on the solubility of the tau aggregates. Insoluble tau fractions possess a much higher seeding capacity compared with soluble fractions. Interestingly, upon injection of tau aggregates into non-transgenic wild-type mice, aggregates have been confirmed as being localized to the injection site. In contrast, after injection into tau transgenic animals, aggregates develop not only at the injection site but spread to anatomically connected brain regions mirroring the highly predictable histopathological pattern of disease dissemination observed in AD patients (Clavaguera et al. 2009). Taken together, these findings indicate that tau aggregation in host animals can be induced by exogenously administered tau aggregates and that their spreading requires the process of aggregate induction and is not simply caused by passive diffusion from the injection site and endocytosis of aggregates.

Similar to α-synuclein, tau is present in the extracellular space, e.g., in the interstitial fluid of the brain, CSF and cell culture supernatant (Yamada et al. 2011). Tau does not contain a conventional secretion signal, although its release seems to be a physiological process that occurs in the absence of neurodegeneration, since tau has been detected in the interstitial fluid of wild-type mice brains and is abundantly present in the CSF of healthy persons, although CSF concentrations increase dramatically after neuronal damage (Tarawneh and Holtzman 2010; Yamada et al. 2011). Exosome-associated tau has recently been described in culture medium and CSF, indicating an active exocytosis process. However, the intracellular sorting mechanisms for exosomal/microvesicular release are unclear, as is the percentage of extracellular tau that stems from this pathway (Saman et al. 2011). As in the case of α-synuclein, no comparative data regarding the neurotoxicity and seeding capacity of free and EMV-associated tau have been obtained so far.

Exosomes from M1C cells contain tau and are enriched in dimeric and trimeric tau species and in threonine 181 phosphorylated tau (Saman et al. 2011). This supports the hypothesis that exosomes might carry oligomeric species that serve as a template or seed to induce aggregation in recipient cells. Whether oligomerization occurs within the exosome, promoted by the high local protein concentration and pH, or whether oligomerized protein is specifically sorted to exosomes, as has been described for membrane-bound proteins (Shen et al. 2011b), is unclear.

Tau protein has been detected by immuno-electron microscopy at the surface of EMVs derived from M1C cells and human CSF; however, these results have to be considered with caution, since the CSF was obtained postmortem and could therefore have contained intracellular vesicles and organelles that were derived from dying cells and that would be co-purified with the EMV fraction (Saman et al. 2011). Because of the topology of EMVs, the cytosolic protein tau would be expected to reside within the vesicle and not at the outer vesicle membrane. The reported results could therefore indicate that tau is leaking from degrading cells and attaches to the EMV surface within the extracellular space, rather than during EMV biogenesis. In order to answer the question of whether tau might also be present within EMVs, further studies are required, including proteinase K digestion or immune electron microscopy of EMV preparations after detergent solubilization. The demonstration of intravesicular tau would indicate an active packaging and secretion pathway, rather than extracellular binding to the surface.

Nevertheless, even by extracellular association with the EMV membrane, tau could be delivered into neurons and contribute to disease spreading. In this context, speculation that the beneficial effect of tau-directed immunotherapy approaches in transgenic mouse models relies on the antibody-mediated targeting of extracellular free and EMV-bound tau species is tempting, since an explanation as to how antibodies could enter the cytosol and be directed against intracellular tau aggregates is not readily forthcoming (Asuni et al. 2007; Boutajangout et al. 2011; Sigurdsson 2009).

Based on the temporospatial progress of tau pathology in AD, Braak and Del Tredici (2011) have proposed a sequential pathway of neurofibrillary tangle propagation affecting the brainstem/locus coeruleus, transentorhinal cortex, neocortical association area and primary and secondary cortical areas and followed by the so-called “return pathway” of corticocortical projections to primary cortical fields. The hierarchical vulnerability of scarcely affected layer IV pyramidal neurons compared with heavily affected layer Va pyramidal neurons has remained enigmatic so far, since it could not be explained by the differential vulnerability of the various cell types. The hypothesis of trans-synaptic transmission, however, could explain the observed sparing of pyramidal neurons in layer IV; these are rarely targeted by the projections from the return pathway (Braak and Del Tredici 2011). The anatomical distance between the locus coeruleus and cortical neurons additionally suggests a pre- to postsynaptic transmission of tau pathology similar to the case of experimental transmissible mink encephalopathy, a prion disease of the mink, in which the retrograde spreading of PrP^sc^ along the sciatic nerve and the spinal cord to the brain stem has been observed in a Syrian hamster model (Bartz et al. 2002). However, this route of transport rather indicates trans-synaptic spreading from the post- to presynaptic sites. Whether tau-positive EMVs are indeed released at the presynapse is unknown, as are the sites of uptake (dendritic/somatic) in recipient cells.

### Amyloid-beta

Intracerebral injection of human AD brain extracts or extracts prepared from human amyloid precursor protein (APP) transgenic mouse brains containing aggregated amyloid-β into the brains of APP transgenic mice induces the formation of amyloid-β plaques in the host brains (Eisele et al. 2010; Meyer-Luehmann et al. 2006). A seeding mechanism is likely, since the immunodepletion of amyloid-β or denaturation by formic acid abolishes the capacity of extracts to induce plaque formation. Furthermore, the induction of amyloid-β deposition requires the combination of human APP transgenic host mice and human APP-derived amyloid-β assemblies in the extract. These experiments also indicate the possibility of interneuronal cell-autonomous disease propagation, since the induction of amyloid-β deposits is not restricted to the injection site but includes axonally connected areas that are not adjacent to each other (Eisele et al. 2010; Meyer-Luehmann et al. 2006). The concept of disease spreading is further supported by the finding that the intraperitoneal administration of brain extracts is sufficient to trigger amyloid-β aggregation in the APP transgenic mouse brain (Eisele et al. 2010). Initiation of rapid amyloid-β assembly by exogenous seeds containing amyloid-β aggregates has also been described after inoculation into the brains of primates (Baker et al. 1994).

In vitro assembled aggregates of either synthetic amyloid-β 40 or 42 fail to induce seeding and a so far unknown co-factor is probably required to induce misfolding into aggregates with seeding properties (Meyer-Luehmann et al. 2006). Interestingly, EMVs carry proteins involved in the generation of amyloid-β (Sharples et al. 2008). APP is cleaved by the sequential action of two secretases (β -and γ-secretase, which release amyloid-β from APP). β-Secretase cleavage produces the APP C-terminal fragment (CTF-β), which can be further processed by γ-secretase (presenilin complex) to APP CTF-γ and amyloid-β peptide. EMV preparations contain full-length APP and CTFs (Sharples et al. 2008). The implications of these findings on APP processing are not clear and whether APP or APP CTF cleavage occurs within the exosome/microvesicle membrane is unknown. A small portion of about 1 %–2 % of total extracellular amyloid-β peptide in the medium of the neuronal cell line N2a has been found to be attached to the surface of exosomes (Rajendran et al. 2006). The exosomal surface could serve as a seed to induce a conformational shift, thereby triggering amyloid-β aggregation. In addition, exosomes could carry amyloid-β peptides to other neurons. However, the impact on oligomerization and interneuronal spreading of amyloid-β pathology clearly needs further investigation.

### Superoxide dismutase 1

In amyotrophic lateral sclerosis (ALS), aggregates of misfolded superoxide dismutase 1 (SOD1) propagate in a spatiotemporal manner linking upper and lower motor neurons (Ravits and La Spada 2009). SOD1 or TDP43 (TAR DNA-binding protein 43) inclusions are the two most common neuropathological hallmarks of the disease (Lagier-Tourenne and Cleveland 2009). The export of misfolded SOD1 and uptake into recipient cells have been shown in vitro (Urushitani et al. 2008). Aggregation of endogenous SOD1 can be induced in cell culture by the exogenous addition of misfolded SOD1 seeds and this templating process continues even after removal of the seed from the culture medium (Grad et al. 2011). Munch et al. (2011) have subsequently been able to demonstrate the interneuronal transfer of SOD1 between cultured cells and the induction of SOD1 assembly in target cells. Some evidence for the in vivo transfer of SOD1 between astrocytes and motor neurons has been provided by recent work of Haidet-Phillips et al. (2011). These authors have isolated progenitor cells from ALS autopsy brains and differentiated them into astrocytes. Co-culturing or the addition of this astrocyte-derived medium induces toxicity in exposed mouse motor neuron cultures; this can be alleviated upon short interfering RNA (siRNA)-mediated SOD1 downregulation in the astrocytes. As has previously been shown in stable motor-neuron-like cell lines expressing wild-type or various SOD1 mutants, SOD1 is at least partially secreted together with EMVs (Gomes et al. 2007). Similar to tau and α-synuclein, experimental data on the toxicity, transfer efficiency and seeding capacity of EMV versus membrane-free SOD1 are lacking.

### Cross-seeding

Cross-seeding between amyloid-β and α-synuclein, α-synuclein and tau, or prion and amyloid-β has been reported in vitro. Indeed, an overlap of disease pathology has often been seen at the histopathological level, e.g. α-synuclein aggregates in AD or tau in Lewy body dementia (LBD). In addition, tau pathology has been genetically linked to PD and LBD. Since both α-synuclein and tau have been detected in EMVs (although definitive evidence that they are present in the same vesicle is absent), these vesicles might represent the site in which cross-seeding occurs.

## Open questions

### In vivo significance and regulation of EMV release

In vivo evidence is needed to answer the question of whether EMVs do indeed confer toxicity and induce seeding in animal models, as has been shown in SAA amyloidosis. The study of the in vivo significance of EMV-mediated disease propagation is hampered by the lack of specific agents to interfere with EMV release or uptake; such agents would enable in vivo studies on the spread of disease pathology. The cell biology of protein sorting and EMV release is still not resolved. An interaction with the endosomal sorting complex required for transport (ESCRT) machinery has been described for mono-ubiquitinated transmembrane proteins; this machinery regulates their sorting into ILVs. Ubiquitin-interacting motifs mediate the binding of ESCRT 0 to cargo destined for sorting into MVBs. The bound cargo is sequentially transported to ESCRT complexes I and II at the late endosomal membrane from where invagination and fission into the endosomal lumen occurs with the help of ESCRT-III (Henne et al. 2011). In contrast, the intra-endosomal budding of other proteins, such as the proteolipid protein PLP, occurs independently of the ESCRT machinery and requires ceramide (Trajkovic et al. 2008). Cytosolic proteins can be sorted into exosomes by their association with lipids and/or transmembrane proteins at the MVE surface or plasma membrane microdomains destined for outward budding. In light of the putative role of EMVs in the pathogenesis of aggregopathies, interestingly, higher-order oligomerization induced by antibody-mediated cross-linking promotes the microvesicular release of various transmembrane proteins such as transferrin-receptor, MHC-I and CD43 (Muntasell et al. 2007; Vidal et al. 1997). Furthermore, the introduction of oligomerization domains to a membrane localization sequence is sufficient to induce ESCRT-independent exosomal release (Fang et al. 2007). The tetraspanin CD63 governs another sorting mechanism into MVEs, a mechanism that is independent of ESCRT and ceramide (van Niel et al. 2011). Strikingly, CD63-dependent sorting of pigment-cell-specific integral membrane glycoprotein (PMEL) targets the protein from the MVE (premelanosome) membrane into ILVs. Here, PMEL is cleaved by two site-specific proteases into the C-terminal fragment and the luminal domain (Kummer et al. 2009). Cleavage is followed by polymerization into physiological PMEL amyloid fibrils within the MVE/premelanosome. This process is reminiscent of the proposed mechanism of intravesicular amyloid-formation. The viral oncogene latent membrane protein LMP1 is another protein that relies on CD63-dependent sorting into a subtype of ILVs that are characterized by low cholesterol and are exosomally secreted (Verweij et al. 2011).

Exosomes can either be secreted in a constitutive or regulated process. An increase in intracellular calcium can trigger MVE fusion and exosome release in various cell types, including neurons, via a mechanism similar to that described for secretory lysosomes (Faure et al. 2006; Savina et al. 2003). The latter process requires synaptotagmin VII, rab27, Munc13-4, AP3 and VAMP7 (Lakkaraju and Rodriguez-Boulan 2008). However, whether these molecules are also involved in MVE fusion and subsequent exosome release is unclear. The secretion of exosomes involves tethering, docking and fusion of the MVE at the plasma membrane. Several regulatory factors of this machinery have been identified, including rab11, the rhoA effector citron kinase, rab27 and rab35 (Loomis et al. 2006; Savina et al. 2002; Ostrowski et al. 2010; Hsu et al. 2010). Calcium enhances exosome release probably by stimulating the fusion of MVEs with the plasma cell membrane in a V-ATPase V0-subunit-dependent manner (Liegeois et al. 2006; Marshansky and Futai 2008). Changes in intracellular ion concentrations after the P2X7-receptor-induced activation of the ATP-gated ion channel have been described to trigger the release of exosomes in immune cells (Qu and Dubyak 2009). Other stimulatory factors, such as DNA damage and (oxidative) stress also promote exosome release, consistent with a role for exosomes in the removal of toxic molecules from the cell (Lespagnol et al. 2008).

Microparticles shed from the plasma membrane are dependent on the calcium-induced reorganization of the cytoskeleton and membrane lipid asymmetry. The outer membrane leaflet of microparticles is enriched in aminophospholipids such as phoshatidylserine (PS) and phosphatidylethanolamine (PE) and the asymmetric distribution of these lipids has been proposed as a mechanism to trigger membrane bending because of their conical shape (Basse et al. 1993; Wehman et al. 2011). Lipid asymmetry is, among other factors, created by the enzymatic activity of scramblase, which translocates and enriches PS and PE from the inner to the outer membrane leaflet (Contreras et al. 2010). This is illustrated by the deficiency of procoagulatory platelet microvesiculation observed in Scott’s syndrome in which the lipid asymmetry of the outer plasma membrane is dysregulated and PE and PS are mainly restricted to the inner leaflet of the bilayer (Lhermusier et al. 2011). Recently, the transmembrane flippase TAT-5 has been shown, in *Caenorhabditis elegans*, selectively to enrich PE within the inner leaflet without affecting PS asymmetry (Wehman et al. 2011). A deficiency in TAT-5 results in PE enrichment within the outer leaflet and vesicle shedding, whereas TAT-1 mutations, which lead to the accumulation of PS within the outer leaflet, have no impact on vesicle release. In addition, Wehman et al. (2011) have identified rab11 and the ESCRT complex as promoting microvesicle formation. Whether the conical shape of PE mediates the outward bending or whether the relative decrease of PE at the inner leaflet shifts the net charge in favour of the anionic PS, which could enhance ESCRT binding followed by vesiculation of the membrane, remains unclear (Wehman et al. 2011).

### Microglial clearance and target cell selectivity

Exosomes can transport obsolete cellular content out of the cell (Pan et al. 1985). This has led to the assumption that the primary function of exosomes might be the disposal of cellular debris and toxic molecules as an alternative to lysosomal processing in cells with low degradative capacity. In the lipid storage disorder Niemann-Pick type C, exosomal release is upregulated and contributes to shuttling excess cholesterol out of the cells (Strauss et al. 2010). Other examples include the shedding of microvesicles to remove complement attack complexes from opsonized cells (Pilzer et al. 2005). Cells can handle protein aggregates by interaction with chaperones and by degradation in the proteasome, lysosome or autophagosome. Exosomal release of toxic or aggregated proteins might serve as an alternative pathway for the cell to remove unwanted content, followed by microglia clearance. For example, microglia cells internalize oligodendrocytic EMVs by macropinocytosis in vitro and in vivo and might thereby establish a clearance mechanism (Fitzner et al. 2011; Zhuang et al. 2011). Activated microglia reside next to amyloid plaques and have been extensively discussed in the context of plaque clearance (Jantzen et al. 2002). Microglia dysfunction has been observed in neurodegenerative diseases and either a deficiency of microglia/myeloid cell function and/or an overload of their endocytosis capacities might enable the intraneuronal uptake of EMV-packed aggregates, which finally might result in the spreading of pathology. The ganciclovir-induced ablation of microglia in an APP mouse model has been shown by Grathwohl et al. (2009) to exert no effect on amyloid plaque formation. The authors therefore speculate that microglia might not have a prominent role in amyloid plaque clearance. However, microglia ablation is induced only after the onset of plaque formation. An effect of microglial function on intercellular disease propagation could be studied in seeding experiments in microglia ablated APP mice. It would be interesting to examine whether microglia deficiency can enhance seeding and interneuronal spreading after the intracerebral injection of amyloid-laden brain extracts.

Of note, several tau or alpha-synuclein aggregopathies are not restricted to the neuronal cell type but can start in the glial cell lineage. An EMV-based transfer mechanism is a feasible explanation of these findings. However, in vivo evidence for oligodendroglial/neuronal EMV transfer is still lacking.

**Fig. 2:**
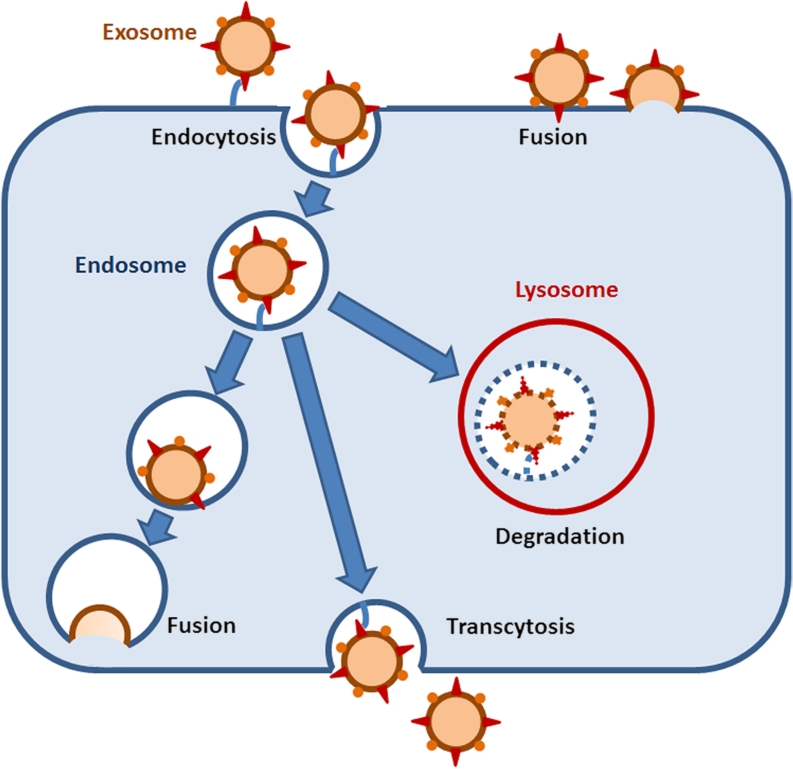
Various modes of exosome entry and intracellular itinery. Exosomes can be internalized by receptor-mediated endocytosis or bulk endocytosis. Once inside the endosome, they can fuse with the endosomal membrane to release their cargo into the cytosol. Alternatively, after fusion of the endosome with the plasma membrane, internalized exosomes can be released into the extracellular space (transcytosis pathway). Fusion of the endosome with lysosomes leads to the degradation of internalized exosomes. An endocytosis-indpendent pathway requires fusion of the exosome/plasma membrane at the cell-surface, followed by release of the exosomal content into the cytosol

This leads to the so far unresolved question of target cell recognition and uptake. Most experiments addressing the transfer and uptake of exosomes into target cells rely on the fluorescence labelling of EMVs prepared by ultracentrifugation in vitro. These exogenously added vesicles tend to form aggregates that might be artificially taken up by phagocytosis and obscure other mechanisms of uptake and interaction. The study of EMV/target cell communication has further been hampered by the fact that single exosomes are below the resolution limit of approximately 200 nm of conventional light microscopy. In a recent study, this obstacle has been overcome in an elegant experiment in which the spontaneous transfer of single exosomes has been monitored by a fluorogenic dequenching assay and has been shown to depend on actin and V-ATPase (Montecalvo et al. 2011). Previous experiments have identified a variety of mechanisms of EMV/target cell interaction including endocytosis mediated by ligand/adhesion molecule binding at the plasma membrane of recipient cells, e.g. VLA-4, alphaM integrin, beta2 integrin (Nolte-’t Hoen et al. 2009; Segura et al. 2005, 2007; Fig. 2). EMVs can also be recognized by the phosphatidylserine cell surface receptor Tim (T-cell immunoglobulin-containing and mucin-domain containing molecule) family transmembrane proteins. Tim1 and Tim4 have been shown to bind to phosphatidylserine present on the EMV surface (Miyanishi et al. 2007; Park et al. 2007). EMVs can be internalized by receptor-mediated or bulk endocytosis, phagocytosis (upon binding of exosomal galectin-5 to membrane galactosidase) and macropinocytosis (Barres et al. 2010; Thery et al. 2002; Fitzner et al. 2011). Internalized EMVs have been detected in late endosomes of dendritic cells by immunocolocalization (Morelli et al. 2004). In order to reach the cytosol intra-endosomal EMVs need to fuse with the endosomal membrane. Alternatively, EMVs might be degraded after maturation of the late endosome to lysosomes. A different scenario implies the re-release of internalized EMVs after storage in MVEs or recycling exosomes, a process that would allow the transcytosis of EMVs and that might play a role in crossing the blood brain or brain CSF barrier. Another mechanism for releasing MVE content into the recipient cell is the fusion of MVE and the target cell plasma membrane at the cell surface.

### Clinical implications

Infectious prion diseases are characterized by inter-individual disease transfer via the natural environment, whereas prionoid transfer is characterized by intra-individual spreading (Aguzzi and Rajendran 2009). For none of the above-mentioned aggregopathies has an infectious transmission between animals or humans been demonstrated. One exception, however, is SAA amyloidosis among cheetahs, which secrete AA fibrils into their faeces and for which an oral transmission has been reported (Zhang et al. 2008). However, no epidemiological or experimental data so far have suggested that aggregopathies can be transferred from one individual to the other. Without further experimental data, the clinical implications of these findings are still uncertain but nevertheless evoke the question as to whether AD pathology might be transmitted via blood transfusion, organ transplants or surgical instruments (Walker and Jucker 2011).

In light of the observed Lewy body pathology in transplanted fetal neurons in PD, stem-cell-based therapy strategies need to be reconsidered. One possibility of escaping the seeding of pathological aggregation in stem cell grafts would be the use of genetically engineered cells that do not express the aggregating protein.

In addition to their putative contribution to disease pathology, EMVs could be employed as a biomarker or as a therapeutic tool in degenerative diseases. Exosomal proteome or microRNAome (miRNAome) profiling is a common approach in the development of novel diagnostic or prognostic biomarkers, especially in oncology (for reviews, see Mathivanan et al. 2010a, 2012). In a similar fashion, CSF or blood exosomes could serve as a diagnostic tool in aggregopathies, especially since several of the aggregating proteins are associated with EMVs.

The potential of EMVs for the targeted delivery of therapeutic drugs is currently under investigation. This emerging concept has been boosted by a recent publication on the targeted exosomal delivery of siRNA-directed against β-secretase in an Alzheimer mouse model (Alvarez-Erviti et al. 2011). One major obstacle of miRNA, miRNA inhibitors or siRNA as a therapeutic approach in various diseases is the challenge of target tissue specificity. In the above-mentioned example of AD, transfer of the therapeutic substance across the blood-brain barrier has to be ensured. Both can be achieved by exploiting exosomes as a transport vesicle, as they carry a neuron-specific targeting signal (Alvarez-Erviti et al. 2011).
